# Where to from here? Policy and financing of integrated community case management (iCCM) of childhood illness in sub–Saharan Africa

**DOI:** 10.7189/jogh.04.020304

**Published:** 2014-12

**Authors:** Kumanan Rasanathan, Salina Bakshi, Daniela C. Rodriguez, Nicholas P. Oliphant, Troy Jacobs, Neal Brandes, Mark Young

**Affiliations:** 1 UNICEF, New York, NY, USA; 2 Johns Hopkins Bloomberg School of Public Health, Baltimore, MD, USA; 3 USAID, Washington, DC, USA

Integrated community case management of childhood illness (iCCM) is a strategy to equip, train, support and supervise community health workers (CHWs) to assess children and deliver curative interventions in communities [1]. In particular, iCCM includes the delivery of amoxicillin (with use of respiratory timers) for pneumonia, oral rehydration salts and zinc for diarrhoea, and rapid diagnostic tests and artemisinin–based combination therapy for malaria. iCCM may also include screening, referral and treatment for malnutrition, and of newborns with illness. A “community health worker” (CHW) in this context is a health worker that provides health care in the community, with some training in the interventions they deliver (and who may or may not receive monetary payment), but who does not have a formal health professional or paraprofessional certificate or tertiary education degree.

In sub–Saharan Africa, recent years have seen increasing recognition of iCCM as a core strategy to deliver care to children, particularly those with poor access to health facilities, and reduce child mortality, in the context of the drive to achieve the Millennium Development Goals. Twenty–eight countries in sub–Saharan Africa are now the site of delivery of community case management for each of pneumonia, diarrhoea and malaria, albeit at widely differing levels of coverage between countries [2]. Despite this progress, there are significant remaining obstacles to realizing the potential of iCCM to provide effective coverage of interventions for childhood illness at scale and quality. Here we review current trends in policy and financing of iCCM in sub–Saharan Africa to highlight two key issues: sustainable financing of iCCM, particularly from domestic sources, and the integration of iCCM in national health systems. We conclude by providing suggestions for how to move forward on these linked challenges.

## FROM POLICY TO IMPLEMENTATION

Policy development for iCCM in sub–Saharan Africa has proved challenging in many countries [3]. It is, however, no longer the major obstacle as most countries now have some type of written policy supporting delivery of care by CHWs, at least for pneumonia, diarrhoea and malaria [2]. The main challenges lie instead in implementation, with problems across countries in supply of commodities, utilization, scale, quality, financing and monitoring of services. iCCM for newborn care, especially treatment of neonatal sepsis, is an exception to this conclusion, as much also still remains to be done in terms of policy development, including greater consensus among development partners about guidelines and the effective role of CHWs in providing care. Few countries in sub–Saharan Africa have established substantive iCCM newborn policy but there is growing momentum.

Despite the overall progress in policy development for iCCM, challenges seen in implementation can be linked to deficits in iCCM policy–making. Implementation of iCCM in sub–Saharan Africa is as heterogeneous as the prevailing health systems, but common policy–related issues can be identified which provide a partial explanation for difficulties encountered in scale, utilization and financing. In too many countries, iCCM policy development has been mainly limited to technical staff in Ministries of Health and development partner agencies, failing to engage sufficient high–level political commitment and thus leadership at the same time as not involving CHWs themselves and the communities for whom iCCM is designed to provide benefit [3]. Even within Ministries of Health, discussions on iCCM have not necessarily been linked to broader health system policy, including dialogues on human resources for health and health systems financing. It is no coincidence that the countries with the greatest progress in scale and achievement in terms of iCCM, such as Ethiopia, Rwanda and Niger, are also those that have had high–level political champions (often at Ministerial level) and positioned iCCM as a core part of their national health strategies [4–7].

It is also important to recognise that iCCM in sub–Saharan Africa has not developed in a vacuum but with critical contextual influences on implementation. Prior to iCCM, many countries had meaningful experiences with community–based use of some maternal and child health commodities and vaccines through CHWs as well as outreach activities [8–10]. In the 1990s, many of these same countries implemented (and continue to implement) the integrated management of childhood illness (IMCI) approach in facilities. Continued implementation has sometimes occurred in fragmented fashion, even following decreasing support from global development partners. So while iCCM has attempted to address some of the shortcomings of this history, countries implement iCCM in the larger context of their own experience and those of peer countries.

## ENSURING CONTINUING AND LONG–TERM FINANCING FOR iCCM

Financing remains a critical concern for delivery of iCCM services, particularly its sustainability. Funding for iCCM in sub–Saharan Africa is overwhelmingly dependent on development partners, including for core activities such as remuneration of CHWs, commodities and general programme support [2]. In very few countries is external support for iCCM provided as general budget support – mostly it is directly provided for programmes. For many countries, future expansion of iCCM is dependent on what development partners will fund, and only a minority of countries report plans to increase funding for iCCM from domestic resources. With so much of the funding dependent on external sources, the future of iCCM programmes in sub–Saharan Africa seems fragile. Even after expected Global Fund support of US$ 50–100 million and likely support from ministries and bilateral donors, a gap of more than US$ 150 million is anticipated for 2015–2017 (Claire Qureshi, personal communication, 9 October 2014).

A key element of any sustainable solution for this problem in most countries is increased domestic financing of iCCM – which depends on the provision of interventions at community level, including iCCM, being seen as a core delivery channel for child services as part of the national health system. Yet Ministries of Finance have often been excluded from discussions on iCCM policy and financing, partly as a result of lack of high–level engagement in Ministries of Health [3]. The dearth of data on the true costs, both actual and marginal, of iCCM, and whether there is any viable alternative to which to compare cost–effectiveness, has also stifled the ability to make the case for greater domestic funding [2].

An iCCM funding issue most prevalent in West Africa that needs specific attention is the persistence of user fees and mark–ups on commodities [2]. This is a clear issue for utilization and equity of iCCM services, given the increasing evidence of the impact of financial barriers to services, particularly on the poorest [11–12]. Addressing this issue requires efforts aligned to broader health financing reform to avoid unintended negative consequences for communities and CHWs and ensure the financial sustainability of iCCM programmes [13].

## INTEGRATING iCCM INTO NATIONAL HEALTH SYSTEMS UNDER COUNTRY OWNERSHIP

The need to integrate iCCM into national health systems is not as self–evident to all as it may appear. But without such integration, persistent obstacles in supply of commodities, sustaining funding, providing adequate supervision, scaling up implementation and monitoring outcomes are unlikely to be overcome – notwithstanding that integration by itself will not resolve all of the problems seen in the functioning and governance of health systems. Yet partly due to iCCM services in most countries in sub–Saharan Africa being strongly driven (and often provided) by development partners, only 9 countries have budget lines in the national health budget for iCCM [2]. There are encouraging trends in the reporting of and supervision of iCCM services to and by health facilities, but in many countries iCCM activities continue to function almost as stand–alone programmes.

This lack of integration of iCCM into national health systems and plans is related to poor integration of CHWs in general. Over thirty years after the Declaration of Alma Ata [14], the role of CHWs remains contested, particularly in terms of whether they should provide curative care. CHWs are often an after–thought in health policy discussions, despite the vital roles they have continued to play even before the advent of iCCM [8]. Countries which retain a strong influence of the primary health care approach have perhaps, unsurprisingly, benefitted from a stronger basis for the development and implementation of iCCM policy, even though lack of coordination and transition between older CHW cadres deriving from the Alma Ata era and newer cadres created for iCCM programmes has been problematic in some countries [3]. A further challenge for the integration of iCCM in national health systems is that given the dependence on development partner resources, local and national health officials may harbour fears that once external support ends, district fiscal resources will be insufficient to continue to provide services – making them hesitant to fully embrace integration.

Ethiopia provides a striking example of a country where iCCM and CHWs are fully integrated into national health systems, plans and budgets. Key to Ethiopia’s relative success has been strong country ownership of iCCM, with the Ministry of Health fully committed to leading the planning process to develop an integrated national plan [4]. In Ethiopia, CHWs have been seen as integral parts of the health system for some time, with health extension workers and a community delivery platform established prior to iCCM being introduced. In this context, CHWs have become part of an evolving primary care unit that is continually adapting to changing circumstances.

In addition to promising examples like Ethiopia, there are other positive signs for integration of iCCM into national health systems. The Global Fund has committed to providing greater funding for iCCM, providing a stimulus for integration of malaria control programmes with care for pneumonia and diarrhoea – which has often proved difficult where strong vertical malaria programmes are established. Memoranda of understanding between the Global Fund and other agencies such as UNICEF also provide the opportunity for a more harmonized approach between development partners towards increasing effective coverage of essential child services.

## WAY FORWARD: OPPORTUNITIES AND CHALLENGES

iCCM shows both the potential and the challenges for health service delivery in communities to extend effective coverage of essential child health interventions. Addressing the two highlighted issues of sustainable financing and integration is crucial to building on the momentum for iCCM seen in recent years to accelerate its contribution to the goal of ending preventable child deaths within a generation [15,16].

A number of key actions to overcome these problems can be articulated, but these need to be adapted to the specific context of each country for iCCM, which varies greatly between and even within countries. First, there is a need for a health systems approach to iCCM to be adopted [17]. Without institutionalizing iCCM as a core part of national health systems, plans and budgets, it will remain dependent and driven by development partners, and vulnerable to changing funding and policy winds. iCCM cannot continue to be discussed and implemented as a separate concern to broader dialogues on human resources for health and health financing within countries, and the “iCCM community” needs to reach outward to engage decision–makers and implementers across the broader health sector and beyond, including Ministries of Finance. This observation applies not only to advocates for iCCM within Ministries of Health and in national contexts, but also to those in development partners working at the “global” level.

**Figure:**
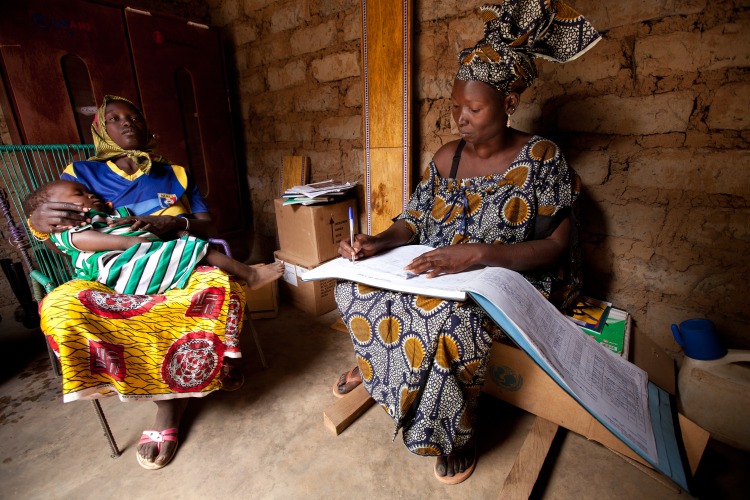
Photo: Courtesy of Michael Crook, UNICEF (©UNICEF/NYHQ2011/MICHAEL CROOK)

Second, the often abstract concept of “sustainable financing” for iCCM needs to be tackled fully and unpacked, again with more granularity for the widely differing contexts in which iCCM is being implemented. Recent progress on costing of iCCM [18], along with the new opportunities provided by the Global Fund, can be built upon. But there is a need for a frank conversation on what part domestic financing can and should play in underwriting iCCM services. In the context of robust and continued economic growth in many sub–Saharan African countries, there is renewed discussion of how domestic health spending can and should rise, and how African countries might meet the call of the Abuja Declaration [19] in terms of the proportion of their national budgets devoted to health spending. iCCM needs positioning by Ministries of Health and development partners to take advantage of these developments.

Third, a prerequisite for discussions on financing and integration is greater clarity on the role of iCCM and whether this is the same for all countries. There is confusion as to whether iCCM is a “stop–gap” measure while countries develop health facilities to provide effective coverage for all children, or whether community–based delivery by CHWs should be seen as a priority and permanent mode of providing services. This debate draws strong emotions, but the direction decided upon needs to be made explicit to underpin any national strategy, as it has important implications for how integration should be undertaken, how CHW cadres should be developed and supported (including in terms of career paths), and how financing decisions should be made. It is difficult to see how most sub–Saharan African countries can achieve and maintain universal coverage of essential child health services without ongoing and institutionalized support for iCCM provided by CHWs, particularly when considering demographic projections of rapidly increasing populations of children under 5 years [20]. But if the claim is made that iCCM is a “stop–gap”, answers are required on what the eventual alternative will be and how this will be financed and implemented. Regardless, a “one–size fits all” approach to iCCM policy and implementation is increasingly insufficient, and a minimum taxonomy of countries is required that provides differential approaches for countries with increasing fiscal space for health and those whom are likely to be dependent on development partners for the foreseeable future.

For the former group of countries, where domestic financing can and should increase (and is doing so) as a proportion of funding for health services, the universal health coverage (UHC) dialogue, including as part of the discussions on the Sustainable Development Goals (SDGs), provides an important policy entry point for institutionalizing iCCM. Efforts to achieve UHC must prioritize improving the provision of essential life–saving interventions for children with poor existing access and coverage – as it would be nonsensical for a country to claim it is moving towards UHC without demonstrating this. Country commitments to achieve UHC are an opportunity to position iCCM as a key strategy to do so– as how else will the communities be reached for which iCCM provides services? Yet, currently there often seems a complete disconnection between UHC discussions and those on iCCM, similarly to how CHW discussions are often marginal in the debates on health workforces.

For the second group of countries, where external resources from development partners will need to fund the bulk of health expenditure for the medium term at least, an alternative strategy is required. UHC remains a distant goal for this shrinking but important group of countries, which includes many with the highest rates of child mortality. Here, equivocations on “sustainable financing” need to give way to explicit commitments by development partners of continued funding and coordinated, ongoing support for the delivery of essential health services for children, prioritising community–based delivery by CHWs, including iCCM, as the basis of national health systems. The current Ebola crisis [21] starkly illustrates the need for this change in approach for this group of countries and is illustrative of the array of system disruptions in these contexts.

In conclusion, further scale–up of iCCM is an opportunity to better integrate community service delivery into national health systems. Recent initiatives such as the aforementioned efforts by the Global Fund, and the recently announced Global Financing Facility [22], provide the opportunity to consolidate a financing basis for iCCM, but further work is required, including mobilization of increased resources and better harmonization to reduce the burden for countries in applying for, receiving and monitoring the use of funding. Moreover, for most countries, the role of domestic financing needs to be clarified and increased. The challenges posed by the increasing demand for universal health coverage, the tragedy of the Ebola crisis and the demographic projections of burgeoning child populations in Africa underline the urgent importance of getting iCCM policy and financing right.
